# A peptide derived from apoptin inhibits glioma growth

**DOI:** 10.18632/oncotarget.16094

**Published:** 2017-03-10

**Authors:** Liqiu Zhang, Hengyu Zhao, Zhongqi Cui, Yueshan Lv, Wenjia Zhang, Xiaoyu Ma, Jianan Zhang, Banghao Sun, Danyang Zhou, Lijie Yuan

**Affiliations:** ^1^ Department of Biochemistry and Molecular Biology, Daqing Campus, Harbin Medical University, Daqing, Heilongjiang, Daqing, P.R. China; ^2^ Teaching Experiment Center of Biotechnology, Harbin Medical University, Harbin, P.R. China; ^3^ Daqing Oilfield General Hospital, Daqing, P.R. China; ^4^ Department of Immunology, Daqing Campus, Harbin Medical University, Daqing, Heilongjiang, Daqing, P.R. China; ^5^ Beijing Sun Palace Community Health Center, China

**Keywords:** HSP70, apoptin, glioma, structural transformation

## Abstract

Glioblastoma (GBM) is associated with poor prognosis due to its resistance to surgery, irradiation, and conventional chemotherapy. Thus, efficient therapeutic approaches for the treatment of GBM are urgently needed. HSP70 is an antiapoptotic protein that participates in the inhibition of both mitochondrial and membrane receptor apoptosis pathways and is highly expressed in glioma tissues. Here, we investigated a derivative of apoptin; specifically, a chicken anemia viral protein with selective toxicity toward cancer cells that can inhibit hyperactive molecules, including HSP70. Our earlier studies demonstrated that apoptin directly binds to the promoter of HSP70 and inhibits HSP70 transcription, which contributes to HSP70 downregulation. This study provides the first demonstration of the therapeutic potential of an apoptin-derived peptide for the treatment of GBM by identifying the minimal region of the apoptin domain required for interaction with the heat-shock element (HSE). This apoptin-derived peptide (ADP) inhibits glioma cell proliferation and tumor growth as well as exhibits an increased ability to promote apoptosis in GBM cells compared with rapamycin and temozolomide. ADP treatment inhibited xenograft tumor growth and increased the overall health and survival of nude mice implanted with GBM cells. These effects were measured in tumors obtained from cell lines and were observed in both intracranial and subcutaneous xenografts. In conclusion, we provide the first demonstration that ADP has therapeutic potential for the treatment of human GBM. Specifically, this study suggests that ADP is a potent candidate for drug development based on its favorable toxicity and pharmacokinetic profiles as well as its time- and cost-saving benefits.

## INTRODUCTION

Glioblastoma (GBM) is the most common intracranial malignancy and accounts for approximately 50% of all gliomas [1]. These tumors are characterized by aggressive invasiveness, long-distance migration, and neovascularization. GBM is one of the most aggressive cancers and is associated with a median overall survival (OS) of approximately 15 months with the standard of care, specifically surgical resection followed by radiotherapy and temozolomide (TMZ) [2]. More recently, two large randomized phase III trials (AVAglio and RTOG 0825), which aimed to compare anti-angiogenic therapy (bevacizumab) with the standard of care, failed to demonstrate a significant improvement in OS [3–5]. Therefore, developing novel molecular therapies for GBM is still a considerable obstacle for basic and clinical medicine.

The 70-kDa heat shock protein (HSP70) facilitates the folding, assembly, transport, and degradation of biological macromolecules. HSP70 inhibits the release of cytochrome C and thereby prevents procaspase-9 from forming apoptosis bodies, which are highly expressed in tumor tissues and weakly expressed in normal tissues [6]. Based on these characteristics, HSP70 is considered a single-molecule target in tumors. Using clinical samples of GBM and normal para-cancerous tissues, we found that HSP70 expression in cancer tissues is significantly increased compared with that in adjacent tissues, suggesting that HSP70 is useful for glioma diagnosis and for pharmaceutical development [7].

We previously reported that apoptin binds to the molecular chaperone HSP70 and the promoter of HSP70 and inhibits HSP70 transcription [8]. Studies assessing the interaction between proteins and the nucleate identified apoptin fragments containing the hydrophobic amino acids in the leucine-rich stretch (LRS, amino acids 33–46), the leucine nuclear export sequence (NES), the nuclear import sequence with the SH3 domain NSL1 (amino acids 82–88), and specific phosphorylation sites in NSL2 (amino acids 111-121). In addition, we added a TAT sequence on the N terminus of the peptide to serve as a tag.

Apoptin, a 14-kDa viral protein (chicken anemia virus protein-3, VP3), can induce apoptosis in cancer cells without affecting normal cells [9, 10] because of its cellular localization. When apoptin is located in the nucleus, it promotes cell death; otherwise, it has no influence on cell viability [7]. In this study, we found high expression levels of HSP70, known as the putative target of apoptin-derived-peptide (ADP), in human GBM samples and a GBM cell line. We also found that ADP retains the vast majority of the antitumor-specific target capabilities of apoptin. In addition, ADP can strongly inhibit HSP70 transcription in GBM cells and exerts a stronger effect than apoptin in promoting tumor cell apoptosis, reducing tumor growth, and improving animal survival. Thus, our results suggest that the synthetic apoptin-derived peptide has a better anti-tumor effect on GBM. Given that HSP70 has a minimal or no expression in normal cells, ADP appears to be an accurate and ideal drug because of his biological characteristics and selective inhibition of HSP70.

## RESULTS

### HSP70 is highly expressed in glioma tissues and GBM cells

**Figure 1:**
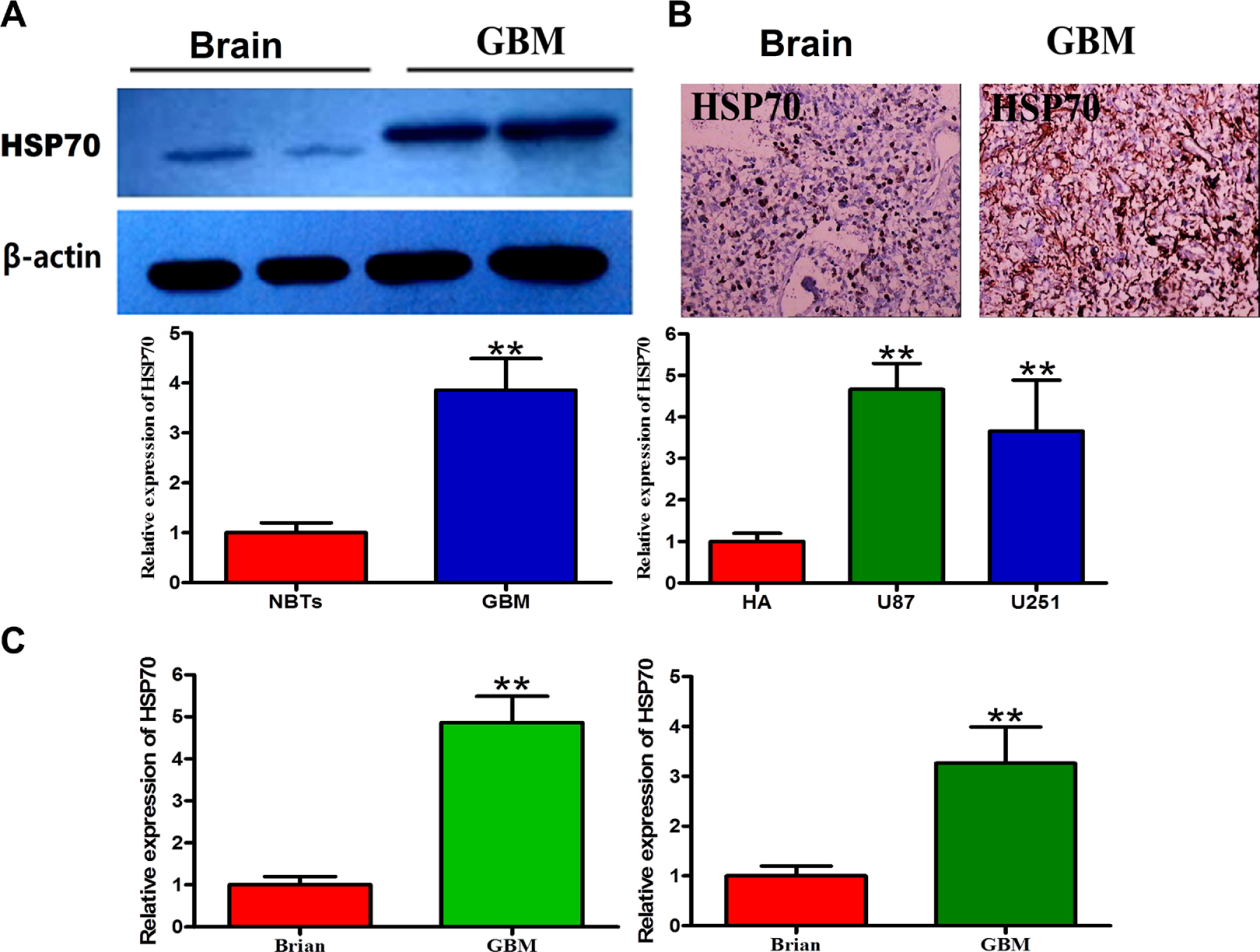
Expression levels of HSP70 in glioma cells and normal cells (**A**) Two representative protein expression patterns in GBM and NBTs obtained through Western blotting using an antibody directed against HSP70. GAPDH was used as an internal reference. (**B**) Two representative staining patterns of GBM and NBTs obtained through immunohistochemistry using an antibody directed against HSP70. NBT was used as a control. (**C**) Comparative analysis of HSP70 mRNA expression in GBM and NBTs.

A Western blot analysis was performed to measure the expression of HSP70 in a series of frozen human GBM samples (Figure 1A), and the results revealed that the expression of HSP70 in GBM was three-fold higher than that in normal brain tissue (NBT). Immunohistochemistry (IHC) staining was performed for authentication, and the results showed that HSP70 expression was significantly increased in gliomas compared with NBT (Figure 1B). HSP70 expression in glioma tissues and GBM cells representing different grades was analyzed by qRT-PCR, and the results showed that HSP70 expression in glioma tissues and GBM cells was significantly increased compared with that in NBT (Figure 1C). We confirmed that HSP70 was expressed in all GBM cell lines, and expression variations were noted among the different cell lines, in accordance with the results obtained for different GBM samples. Therefore, these two GBM cell lines represent appropriate models for the study of the anti-tumoral effects of HSP70 inhibition in GBM.

### Design of an apoptin-derived peptide (ADP)

A screening of the domains of the structure shared between apoptin and the HSE revealed that the induction of HSP70 inhibits GBM cell death. A previous study conducted by our laboratory showed that HSP70 is expressed at lower levels in tumor cells treated with apoptin. Furthermore, apoptin downregulates HSP70 in tumors by binding directly to the HSE, which is the promoter of HSP70, and inhibiting HSP70 expression [11]. Here, we found that both apoptin and ADP can specifically interact with the SRC homology 3 (SH3) domain on the HSE.

**Figure 2:**
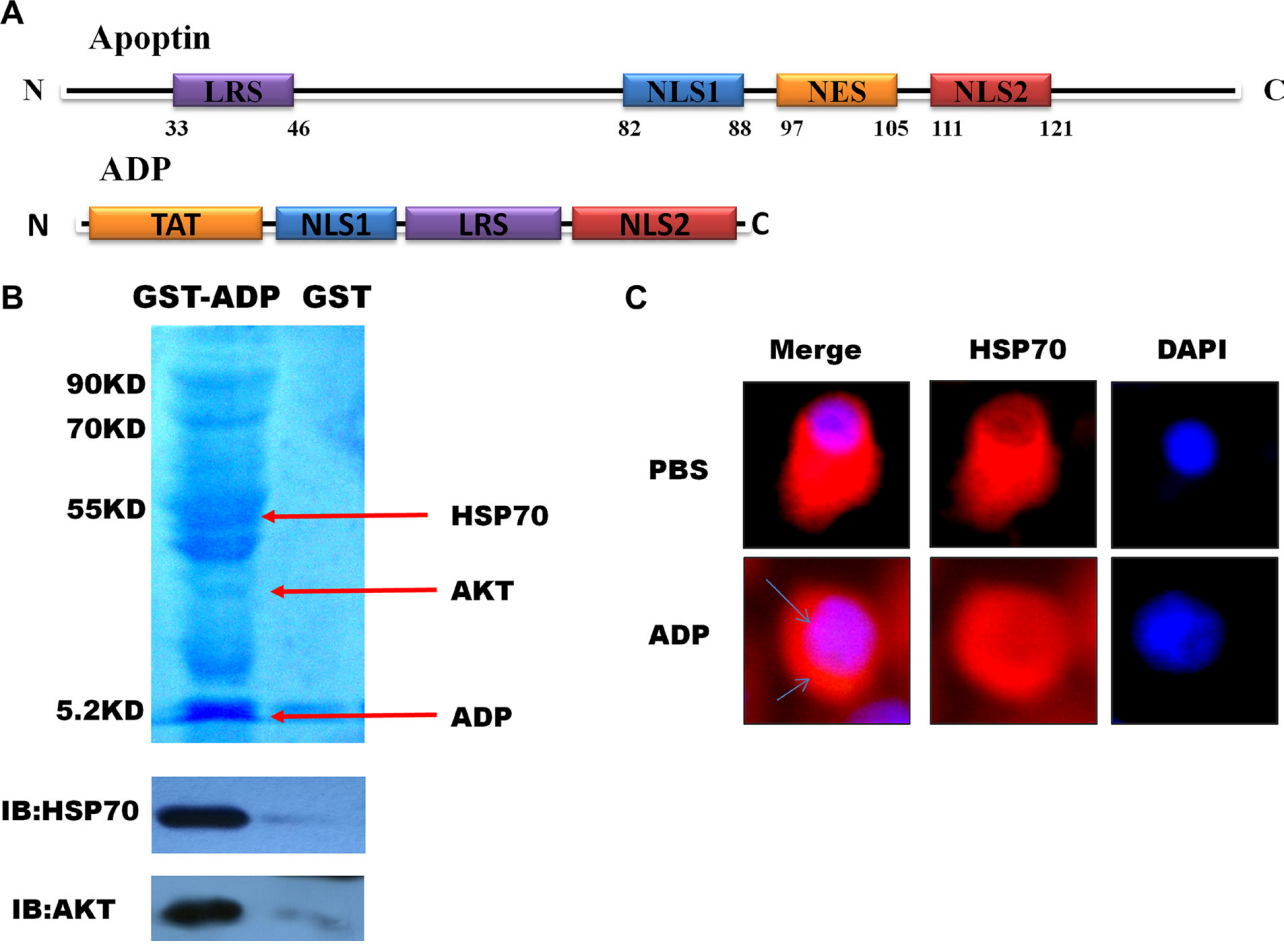
Mapping and modeling of the apoptin motif responsible for its interaction with HSE and HSP70 (**A**) Schematic diagram of apoptin and the apoptin-derived peptide. (**B**). Immunoblot showing the expression of GST-ADP in U87-MG cells and immunoprecipitated with anti-GST antibody 24 hours post-transfection. (**C**). U87-MG cells were treated with ADP for 6 hours, stained with HSP70 antibody followed by secondary antibodies conjugated to Cy3, and then observed under a confocal scanning microscope.

First, we generated several apoptin fragments, which included the LRS, NES, nuclear sequences that contain the SH3 domain NSL1, and specific phosphorylation sites in the NSL2 domain. Our electrophoretic mobility shift assay (EMSA) results indicated that NLS1 and NLS2 strongly interact with the HSE (data not shown) when equal amounts of NSL1 and NSL2 are added simultaneously. NSL1 was found to have a stronger ability to bind to the HSE. In addition, combination strips with the HSE increased significantly after NSL1 and NSL2 were added simultaneously. A combination of NSL1 and NSL2 exhibited stronger binding capacity with the HSE (results not shown). LRS sequences are rich in hydrophobic amino acids. Therefore, we designed a peptide that contained the NLS1 (amino acids 82-88), NLS2 (amino acids 111-121), and LRS structural domain (amino acids 33-46), which is rich in hydrophobic amino acids, from apoptin. We hypothesized that ADP could bind multiple proteins. *In vitro* pull-down assays using U87-MG glioma cell lysates revealed that the ADP polypeptide interacts with a GST tag. A subsequent electrophoresis analysis revealed several protein bands (Figure 2B). To further verify the interaction between the two domains *in vivo*, we performed co-immunoprecipitation assays to detect the interaction between ADP and HSP70, and our results revealed that ADP interacts with HSP70.

To examine the interaction between ADP and HSP70 *in vivo*, we performed an immunofluorescence analysis to assess the impact of apoptin on the subcellular location of HSP70. In U87-MG cells treated with PBS, HSP70 was evenly distributed in the cytoplasm and the nucleus, whereas in U87-MG cells treated with ADP, the HSP70 expression levels were decreased after 6 hours, and the distribution of HSP70 in the cytoplasm decreased (Figure 2C).

### Structural analysis of the apoptin-derived peptide

**Figure 3:**
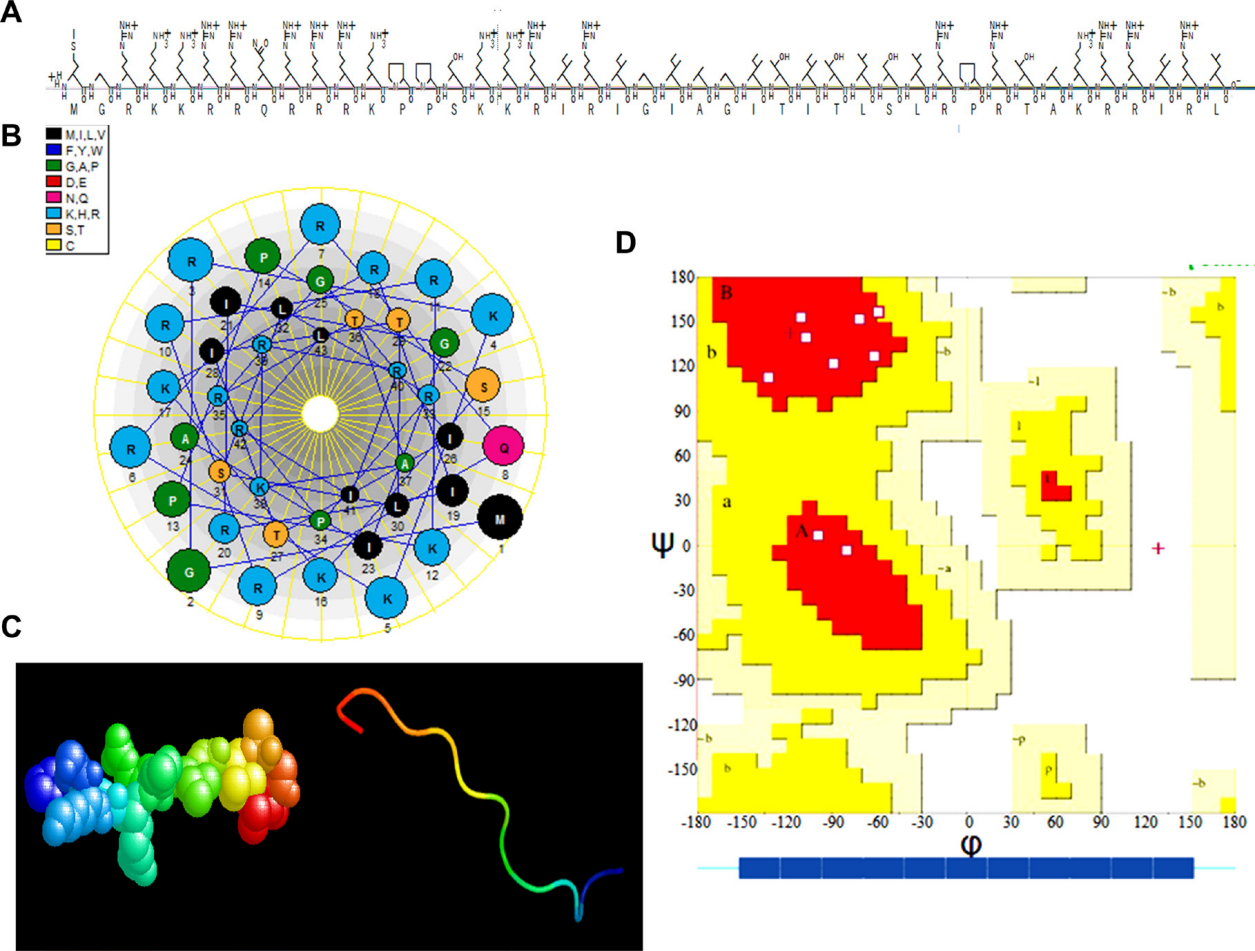
Three-dimensional (3-D) characterization of ADP (**A**) Amino acid sequence and primary structure of ADP. (**B**) Schiffer-Edmundson helical wheel representation of ADP: the helical wheel projection of the arrangement of amino acids and residue numbers are counted from the N terminus of ADP. (**C**) 3-D structure. (**D**) Ramachandran plot analysis of ADP. All non-glycine and proline residues are shown as filled black squares.

The amino acid composition and sequence arrangement of ADP are presented in Figure 3A. The molecular weight of ADP is 5.2 kDa, and its isoelectric point is 13.18. ADP is hydrophilic, exhibits good solubility, and can be dissolved in PBS. Schiffer-Edmundson helical wheel modeling using DNAstar software suggested a possible amphipathic α-helical conformation (Figure 3B). Furthermore, 3D prediction models (PHYRE2 server) (Figure 3C) and Ramachandran plot (UCLA-DOE LAB server) analyses were performed (Figure 3D). The three-dimensional structure of ADP showed an M shape-like structure. The ADP structure domain contains four parts: a penetrating peptide TAT and the core NLS1, LRS, and NLS2 sequences. The LRS structure forms a flexible connection between the two nuclear sequences to effectively maintain the original independent structure, and TAT protects the peptide when entering cells.

### Effect of the apoptin-derived peptide in human glioma cells

**Figure 4:**
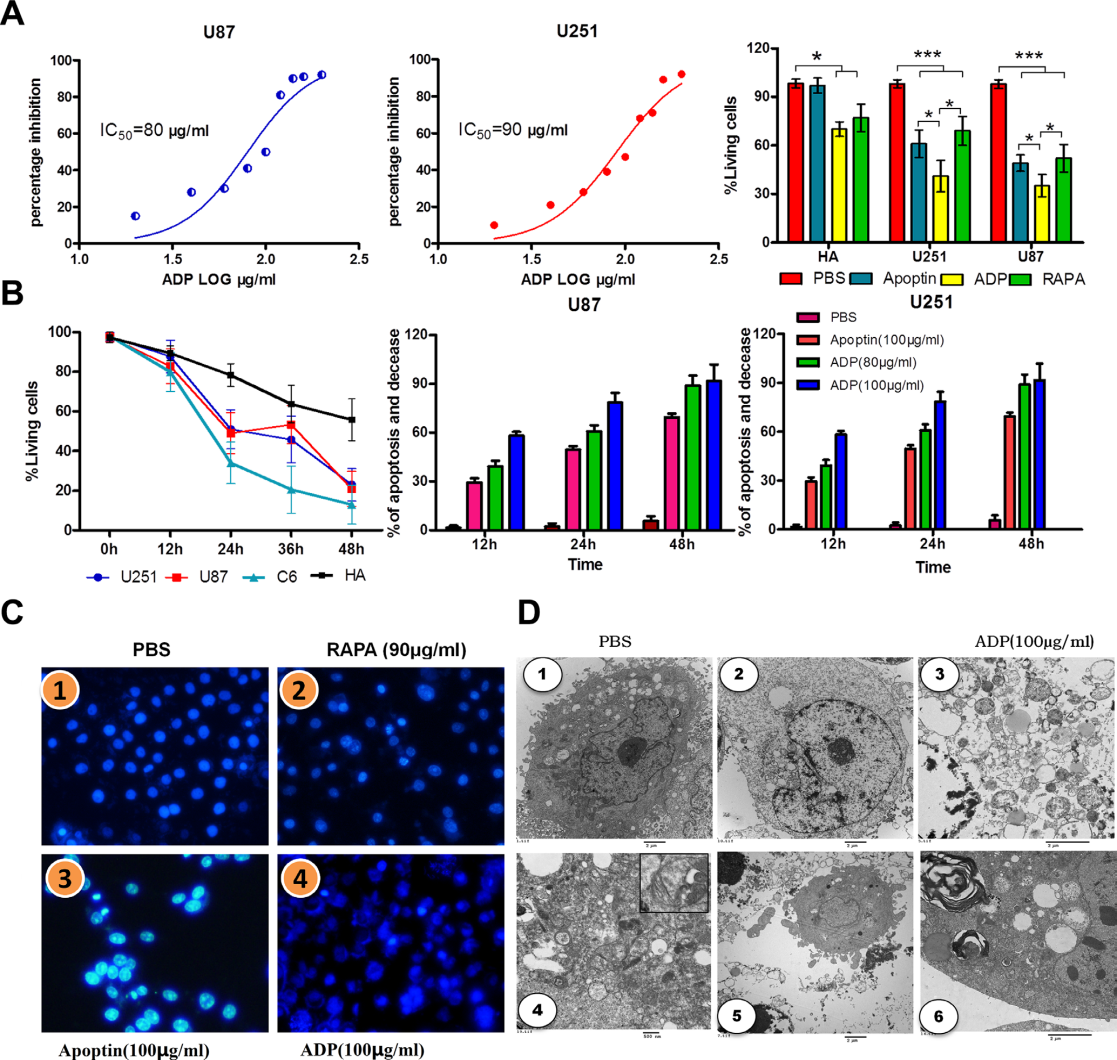
Comparative efficacies of ADP on inhibiting GBM cell growth and its effect on cell viability (**A**) The IC_50_ values were calculated from the % inhibition vs. log ADP concentration curves. (**B**) U251-MG, U87-MG and normal brain cells were treated with 80 and 90 μg/mL ADP for 12, 24, or 48 hours, stained with Annexin V and propidium iodide and then subjected to flow analysis. The two tumor cell lines (U251-MG and U87-MG) demonstrated a greater increase of apoptosis compared with normal brain cells. The MTT assay showed a concentration-response effect of ADP on the growth/viability of GBM cells and normal brain cells. (**C**) The nuclear morphologies of U87 MG cells stained with Hoechst 33342 dye were visualized 24 hours after infection. Scale bar, 4 μm.1 PBS, 2RAPA (90 μg/mL), 3ADP (80 μg/mL), 4ADP (100 μg/mL). (**D**) Transmission electron microscopy of ultrathin sections. U87-MG cells were treated with 100 μg/mL ADP and PBS for 24 hours. 1 PBS; 2-6100 μg/mL ADP.

We evaluated the cytotoxic effect of ADP in the U87-MG and U251-MG GBM cell lines using the MTT assay. The IC_50_ (concentration required to inhibit 50% of cell viability) values were 90 μg/mL for U87-MG cells and 80 μg/mL for U251-MG cells (Figure 4A). The proliferation of the cells grown in the absence of the treatment was considered 100%, and cell survival was expressed as a normalized average. As shown in Figure 4A, after treatment with 80 μg/mL apoptin-derived peptide for 24 hours, the survival rate of glioma cells was significantly reduced compared with that of the control group. This growth rate was also slightly lower than that observed after apoptin treatment and higher than that found after rapamycin (RAPA) treatment (32 μM). Annexin V-FITC flow cytometry was performed to assess U87-MG and U251-MG cells treated with the TAT-apoptin derived peptide, and the level of apoptosis was assessed 24 hours later. RAPA (32 μM) was used as a positive control, and an equal volume of PBS was used as a negative control (Figure 4B).

The results of the apoptosis analysis, which are presented in Figure 4B, revealed that TAT-apoptin-derived peptides (80 μg/mL) promote apoptosis to a greater extent than apoptin in glioma cells. Treatment with ADP for 24 hours resulted in the highest apoptosis level. These results further confirmed that the apoptin-derived peptide, through its interaction with the HSE domain, mediates glioma cell apoptosis. Notably, compared with apoptin, apoptin-derived peptides increased apoptosis in human astrocytes, but the extent of apoptosis remained low compared with that in glioma cells. Thus, we cannot rule out that the apoptin-derived peptide-specific induction of apoptosis does not affect normal cells.

To further analyze the mechanism through which apoptin-derived peptides promote apoptosis in glioma cells and inhibit growth, we performed Hoechst 33342 nuclear staining (Figure 4C). Our results indicate that the cell nuclei in the control group appeared normal, whereas in the apoptin group, glioma nuclei were hyperchromatic, and chromatin was highly condensed and marginalized. In addition, some parts of the nuclei were fragmented, which is indicative of apoptosis. In the apoptin-derived peptide group, the majority of cells were fragmented, and apoptosis was significantly enhanced. The effects of RAPA treatment were similar to those obtained with the treatment with the apoptin-derived peptide. Our results suggest that ADP efficiently induces apoptosis in GBM.

**Figure 5:**
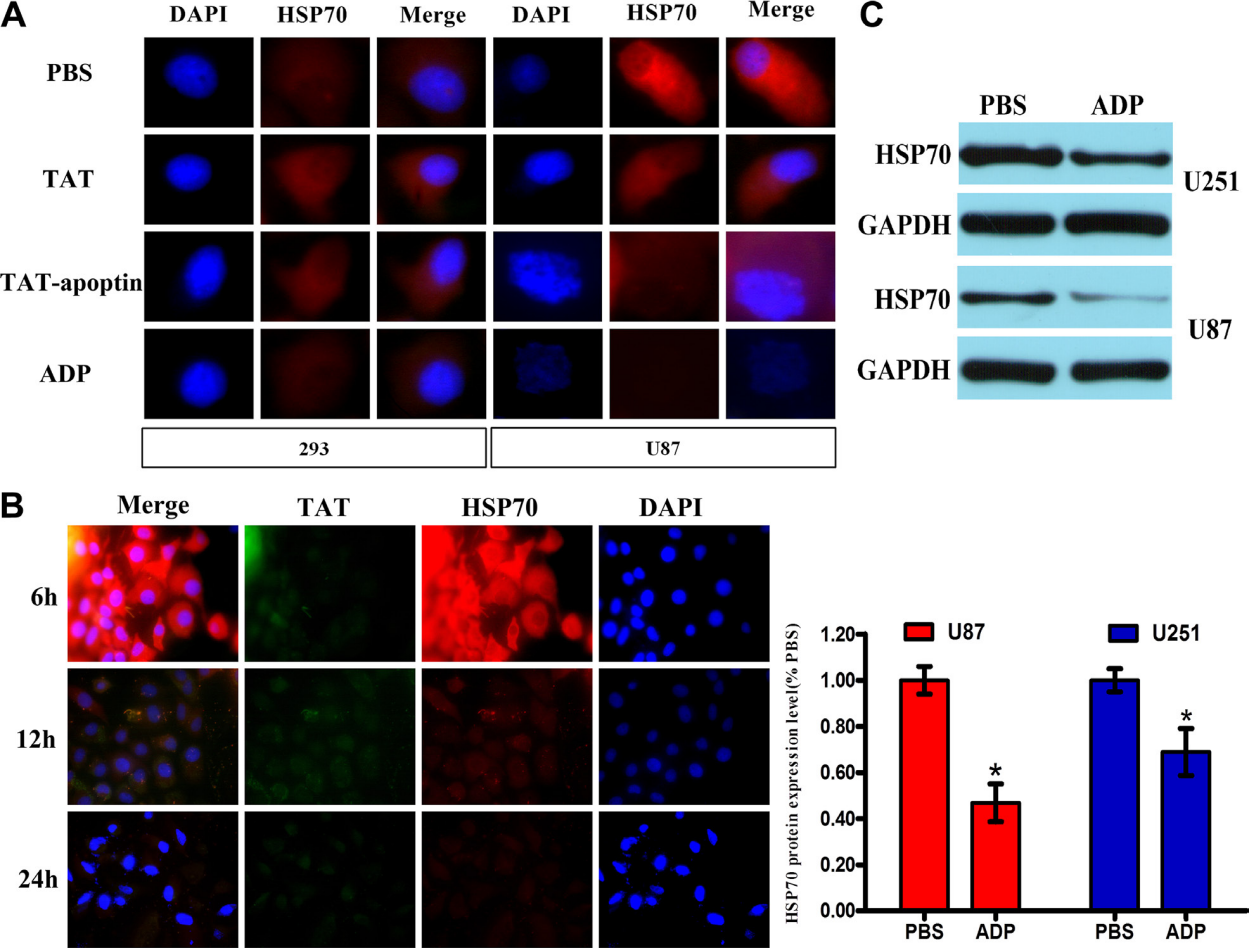
ADP downregulates HSP70 expression in GBM cells (**A**) U87-MG and 293 cells were treated with 80 μg/mL ADP for 24 hours, stained with HSP70 or TAT antibody followed by secondary antibodies conjugated to Cy3, and then observed under a confocal scanning microscope. Bar: 10 μm. (**B**) U87-MG cells were treated with 80 μg/mL ADP for 6, 12 or 24 hours and processed according to above-described analysis method. (**C**) Western blot analysis of the HSP70 levels in U251-MG and U87-MG cells treated with 80 μg/mL ADP. *p < 0.05 versus the control group (PBS).

**Figure 6:**
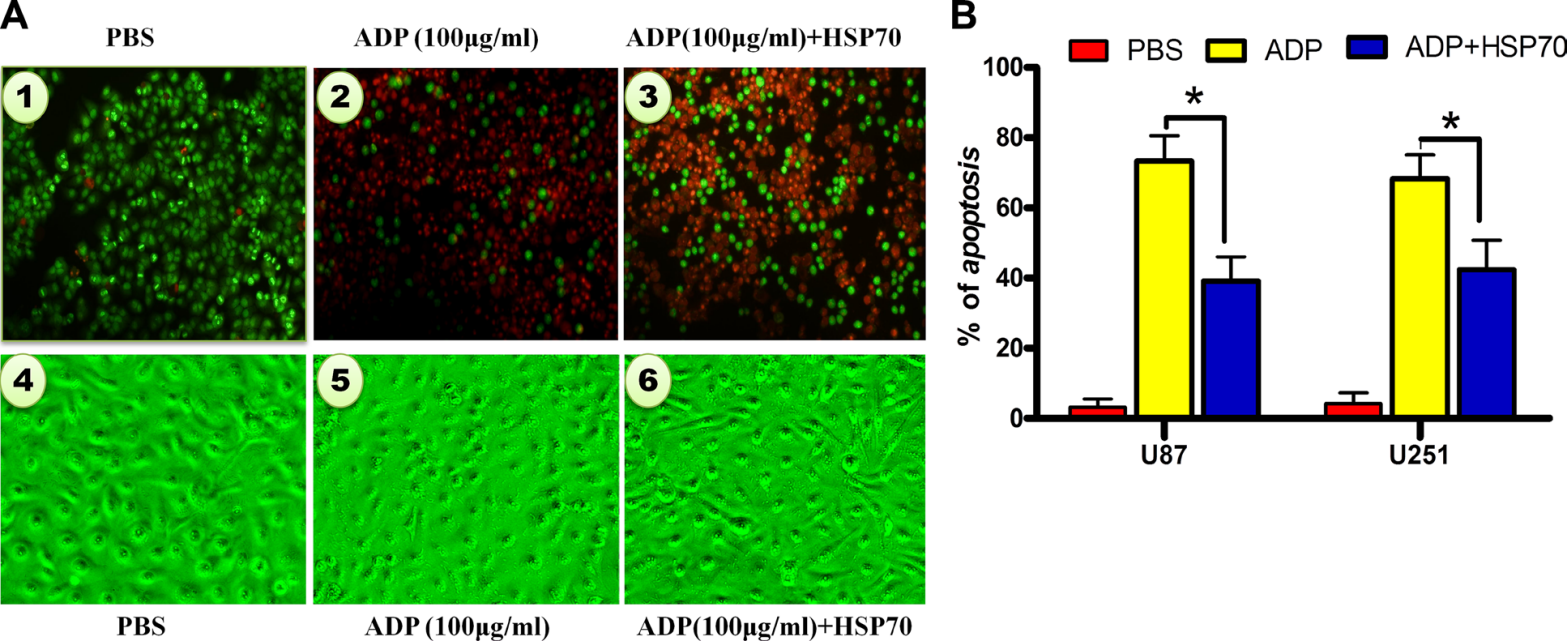
HSP70 antagonizes ADP-induced apoptosis in U87-MG tumor cells (**A**) U87-MG cells were transfected with pcDNA3.1-HSP70 for 4 hours and treated with ADP for 24 hours. The cells were fixed with 3% formaldehyde, stained with AO/EB, and evaluated by microscopy. Representative cells are presented. Bar: 1 μm. (**B**) The cells were treated as noted in (a), and the percentage of apoptosis was calculated by Annexin-V-FITC/PI staining and flow cytometry analysis (*N* = 3).

Electron microscopy of identical tumors showed large amounts of cell debris and liquid droplets scattered within the visual field (Figure 4D). Blurring between edema in the tissue and liquid on the tumor edges is obtained. The amount of cell debris was markedly reduced in cells treated with ADP. Electron microscopy revealed filaments that formed structures mirroring that of other cells to maintain the cell shapes. Between the filaments were small fragments of cell debris. (Figure 4D-4). Actin and intermediate filaments were indicated by structures 7 to 10 nm in thickness. Thus, we discovered that the cytoskeleton of tumor cells presented characteristic changes after ADP administration, which was coincident with shriveling of tumor tissues and mitochondrial autophagy (Figures 4D-5, 6).

### ADP induces glioma cell apoptosis by reducing HSP70 expression

Apoptin induced apoptosis in cancer cells by activating caspases via the intrinsic/mitochondrial death pathway instead of the extrinsic/receptor-based pathway [12]. To further confirm the characteristics of ADP-induced glioma cell death, we compared the nuclear morphology of apoptin/ADP-untreated and apoptin/ADP-treated 293 and U87-MG cells by analyzing the apoptotic nuclei. In addition, we hypothesized that ADP induces apoptosis in glioma cells by reducing HSP70 expression, a key target of cell survival. The results of the Western blotting and immunocytochemistry indicated that HSP70 was expressed (Figure 5A and 5C). In these experiments, the apoptin treatment group showed obvious apoptotic nuclear morphology, indicating that apoptin has a strong ability to induce U-87 cell apoptosis (Figure 5A and 5C). Our results showed that after ADP treatment, HSP70 expression decreased in a time-dependent manner (Figure 5B). In addition, we added an equal amount of ADP to 293 cells and assessed HSP70 expression. Our results demonstrated that the HSP70 levels did not significantly change in 293 cells, indicating that ADP specifically induces apoptosis in tumor cells.

### HSP70 antagonizes ADP-induced apoptosis in tumor cells

To investigate the role of HSP70 in ADP-induced apoptosis in tumor cells, we constructed the HSP70 overexpression plasmid pcDNA3.1-HSP70. After we transfected the plasmid into U87-MG cells. We then employed AO/EB staining to detect ADP-induced apoptosis in U87-MG cells with modulated HSP70 expression levels. Our results indicated that the high levels of HSP70 expression strongly inhibited the proapoptotic effect of ADP. The number of apoptotic cells was decreased remarkably, and the vast majority of cells exhibited a healthy morphology. In contrast, in cells treated with ADP, most of the nuclei were stained an orange-red color, which is indicative of nuclear shrinkage and fragmentation in late apoptotic or dead cells (Figure 6A). We subsequently performed Annexin-V-FITC/PI staining and a flow cytometry analysis of apoptosis. The results revealed that 19.3% of ADP-treated U87-MG cells underwent apoptosis at 24 hours. HSP70 overexpression significantly suppressed ADP-induced apoptosis (Figure 6B). Collectively, these results demonstrate that HSP70 antagonizes ADP-induced apoptosis in tumor cells.

### ADP reduces tumor growth *in vivo*

**Figure 7:**
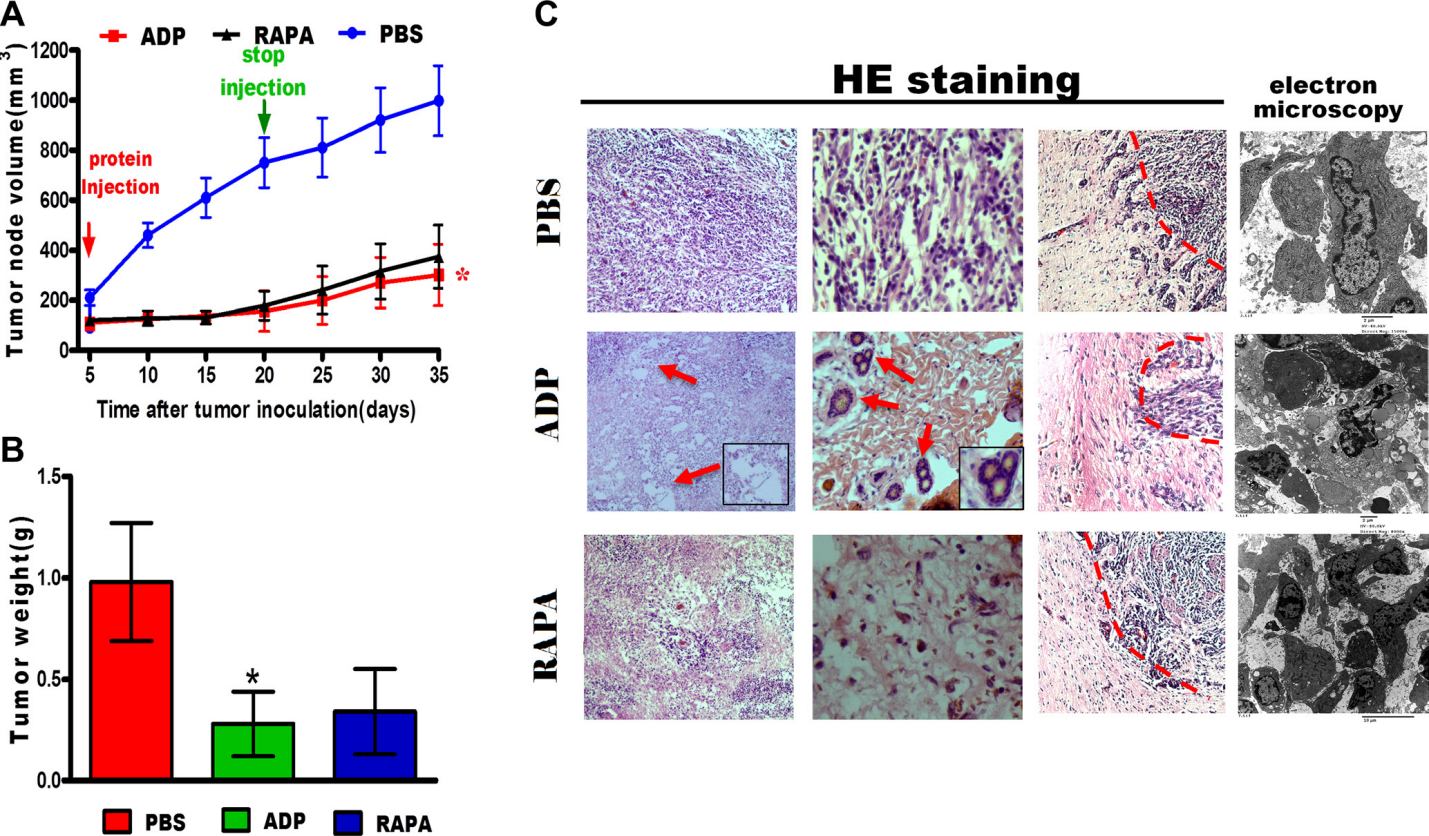
ADP inhibits U87-MG cell growth *in vivo* (**A**) The tumor sizes were measured every two days. At the end of the measurement period, the tumors were excised (*N* = 4; *p < 0.05). (**B**) At the end of the treatment period, the tumor weights were measured (*N* = 4). The data represent the means ± SD of each group (*p < 0.05). (**C**) Tumor tissue sections were subjected to HE staining and electron microscopy analysis (Scale bars: 50 μm).

We constructed a subcutaneous tumor mouse model by injecting U87 cells into the armpits of nude mice. All of the mice were randomly divided into the control group, the apoptin-derived peptide group, and the RAPA group. The tumor volume in all tumor-bearing mice was assessed over 30 consecutive days. The mice were then euthanized, and the tumors were removed. As shown in Figure 7A, tumor growth was significantly inhibited by ADP treatment, as indicated by a significant decrease in tumor volume of 301.9 ± 3121.6 mm^3^ compared with that observed for the control group (998.5 ± 140.2 mm^3^). The tumor volumes increased rapidly in the control group, as shown in Figure 7B. The excised tumors from the control group weighed 0.98 ± 0.29 g, whereas the weights of the tumors from the ADP-treated animals and RAPA-treated animals averaged 0.28 ± 0.16 g and 0.34 ± 0.11 g, respectively.

The tumors were sectioned and stained with hematoxylin/eosin for conventional histology. As expected, the histological examination revealed high cellularity in all U87-MG subcutaneous tumors. Enlarged intercellular spaces were observed in the treated tumors as a hallmark of ADP efficacy (Figure 7C). Accordingly, U87-MG cell proliferation was reduced in the treated tumors, revealing apoptin-mediated tumor efficacy and a loss of cell structure integrity. Moreover, electron microscopy analyses also revealed differences between the treated tumors and the control samples. As expected, the histological examination revealed high cellularity in all U87-MG subcutaneous tumors. Enlarged intercellular spaces were observed in treated tumors, indicating the efficacy of ADP (Figure 7C). Overall, these results demonstrate that ADP has a stronger ability to inhibit tumor cell invasion *in vivo* compared with RAPA.

For U87-MG subcutaneous tumors, the mice were treated with ADP and TMZ or with vehicle from five to 14 days after cell implantation. A total of 12 orthotopic intracranial tumors were generated in nude mice after the stereotactic injection of U87-MG cells. Of the 12 mice, four mice (U87-MG) were treated with ADP, four mice (U87-MG) were treated with TMZ, and four mice were treated with PBS.

**Figure 8:**
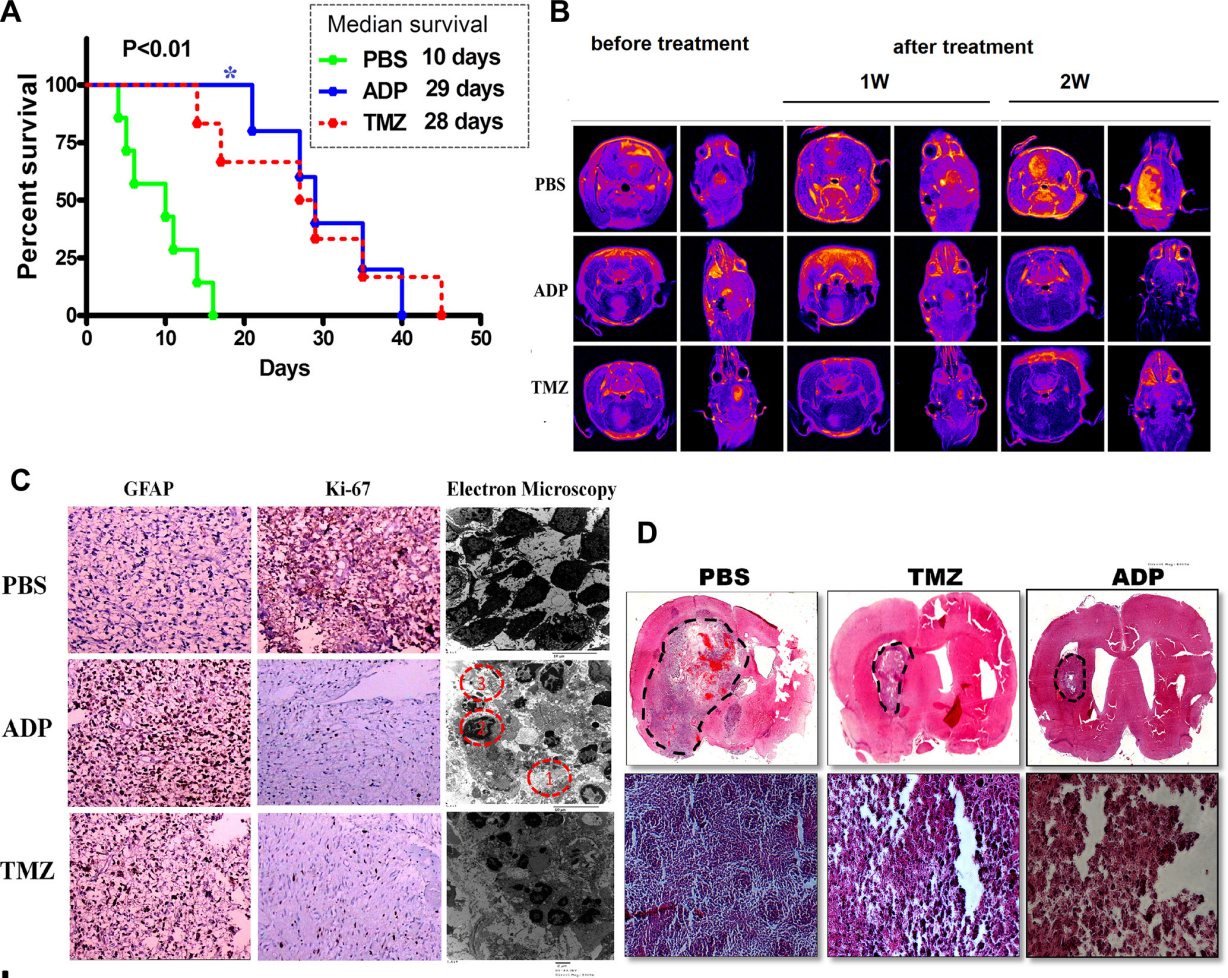
Evaluation of ADP therapeutic effect in an orthotopic glioma model (**A**) Kaplan-Meier survival curves of mice intracerebrally grafted with U87-MG cells and then treated with ADP (5 mg/kg/day, *N* = 3) or TMZ (25 mg/kg/day, *N* = 3). PBS (*N* = 3) served as a control (*p* < 0.05). (**B**) MRI evaluation of the therapeutic effect of ADP in an orthotopic glioma model (*N* = 4). (**C**) Ki-67 and GFAP immunostaining of U87-MG cells treated with ADP or TMZ. PBS was used as a negative control. (**D**) Images of hematoxylin/eosin-stained U87-MG cell tumors treated with ADP or TMZ. Full brain coronal section and magnification revealed infiltrating tumor cells within the corpus callosum. Scale bar (full brain coronal sections, magnification, and hematoxylin/eosin staining): 100 μm.

After the experimental treatments, we extracted the brains of the mice using U-type tweezers and stained the tissues to look for tumors using a microscope. Only mice bearing tumors were included in the survival analyses. Interestingly, ADP treatment induced significant survival benefits compared with vehicle injection (*p* < 0.001 for U87-MG). The median survival was increased more than two-fold in the ADP-treated mice. A similar improvement in median survival was observed in the TMZ-treated mice. The survival curve for the ADP group was essentially flat (Figure 8A).

The apoptin-derived peptide was administered to the mice via tail vein injection, and an abnormal reaction was rarely observed. Dynamic MRI conducted for seven consecutive days revealed tumor foci after ADP administration. We measured the tumor volume using MRI software and performed HE staining after the animals were euthanized. As shown in Figure 8B, the tumor volume of the control group increased over time.

The tumor volume was clearly reduced in the ADP group, as indicated by the lack of detection of these tumors by MRI. Moreover, the relative reduction in tumor volume after TMZ administration was less than that observed in the ADP group. After one week of continuous TMZ administration, the tumor-bearing mice exhibited a loss of appetite and decreased mobility. The treatment and control groups presented statistically significant differences. Conventional histology was analyzed using HE staining and antibodies targeting either Ki-67 or glial fibrillary acidic protein (GFAP).

GBM cell proliferation was reduced in the treated tumors, as demonstrated by decreased Ki-67 staining. Moreover, an increase in GFAP staining in the ADP-treated tumors suggested U87-MG cell differentiation. First, enlargement of the intercellular spaces was noted in the ADP-treated tumors (Figure 8C f(1)). In addition, the treatment induced chromatin aggregation within the nuclei (Figure 8C f(2)) and glial filament accumulation (Figure 8C. f(3)). The apoptotic bodies simultaneously increased, indicating ongoing cell death and differentiation. Interestingly, HE staining indicated that ADP treatment could inhibit glioma cells *in vivo*. Compared with the control group, the ADP group surprisingly showed obvious reductions in both satellite-like cell masses and changes of apoptotic morphology (Figure 8C). Electron microscopy analyses also revealed differences between the treated and control tumors.

The histological analysis of HE-stained brain sections revealed large lobular tumors in the PBS- and ADP-treated groups (Figure 8D). Large tumors were also observed in the PBS-treated mice. The mice treated with PBS presented large numbers of ring-like tumors dispersed around the striatum. We also observed tumors distant from the U-87 cell injection site, and cell invasion was increased along the corpus callosum. In the ADP treatment group, only a few tumor cells were observed near the injection site, and a small number of tumor masses were present at the administration site, demonstrating significant inhibition of tumor mass growth consistent with the MRI findings. All of these results indicated that continuous injections of ADP were the most efficient treatment against GBM.

## DISCUSSION

HSP70 generally exhibits increased expression in GBM tissues compared with normal brain tissues. Endogenous HSP70 protects cancer cells from cell apoptosis, thus conferring resistance to cell death. [13, 14]. Extracellularly, HSP70 is a danger signal that induces antitumor immune responses. Preclinical data have hinted that HSP70 release is significantly increased in glioblastoma cell lines treated with fractionated radiation at single doses of 2 Gy [11]. Intracellularly, increased copy numbers of HSP70 mRNA are correlated with high-grade gliomas. Interestingly, HSP70 expression was found to be significantly increased in GBM compared with NBT. Furthermore, we did not detect HSP70 expression in NBT surrounding the tumor. The available data indicate that HSP70 is expressed in the primary tumors and in relapsed *de novo* GBM patients [7]. An increased expression of extracellular HSP70 was noted in relapsed patients. The significant increase in HSP70 expression observed in relapsed patients might serve as a molecular target of novel therapy approaches [12].

Our previous results showed that TAT-apoptin inhibits HSP70 expression in tumor cells such as liver cancer and gliomas, and the ensuing apoptosis was dependent on the HSP70 expression levels. Furthermore, apoptin inhibited the transcription of HSP70 by binding to the HSE, which downregulated HSP70 expression [8, 15]. We further sought to identify the shortest sequence of apoptin that could promote HSP70 downregulation and mimic the inhibitory activity of apoptin. The current EMSA results showed that NSL1 (amino acids: 82-88) and NSL2 (amino acids: 111-121) strongly interact with the HSE. LRS sequences are plentiful in hydrophobic proteins. Using pull-down assays, we confirmed the interaction between HSP70 and the domain of apoptin corresponding to amino acids 33-46 (LRS). We aimed to increase the ability of apoptin to induce tumor cell apoptosis while reducing the length of its polypeptide sequence. Thus, we removed the NES (amino acids: 97-105) domain and retained the NLS1 and NLS2 domains, which were connected by LRS. The LRS structure forms a flexible connection between the two sequences in the nucleus to effectively maintain the original independent structure. The LRS is consistent with the notion that the β-sheet structure on the surface can effectively interact with HSP70. The TAT (penetrating peptide) sequence was placed in the N-terminal peptide chain. TAT is effective in protecting the apoptin-HSE polypeptide during cellular entry. Furthermore, we tested the activity of ADP in GBM cell lines (U87-MG, U251-MG) as well as in subcutaneous and orthotopic glioma models and found that ADP is an efficient treatment for GBM tumors.

Some apoptosis-related sequences are closely related to the specific binding capacity of tumor cells. For example, the pro-apoptosis activity of apoptin involves the nuclear sequence (NLS), the NES, and the phosphorylation of amino acid 108. The level of specifically targeted tumor apoptosis was decreased slightly by the ADP structure without nuclear sequences and no phosphorylation of amino acid 108 [9, 16, 17]. We hypothesized that ADP would not exhibit the ability to target tumor cells. A portion of normal glial cells were killed in our experiments, but ADP retained the vast majority of the antitumor-specific targeting capabilities of apoptin. When the T108 phosphorylation site is abolished, the nuclear localization of apoptin in tumor cells does not change significantly. [16, 18]. These confounding might be because both the N- and C-terminal domains of apoptin can bind DNA. In addition, phosphorylating (T108) is only required for the C terminus, whereas the N terminus is not affected by tumor-selective phosphokinases [10, 18, 19].

The PI3K/AKT signaling pathway serves an important role in cell survival and cell death processes; different stimuli elicit different effects [20–22]. The toxicity of apoptin depends on hyperactivation of the PI3K-Akt pathway [19, 23], which results in the nuclear translocation of Akt [18]. Akt is normally localized in the cytoplasm but relocates to the nucleus in the presence of apoptin [24]. Nuclear Akt acts as an apoptosis stimulator rather than as a repressor because it can interact with and phosphorylate a different set of substrates in the nucleus [25]. Therefore, apoptin has been proposed to ‘hijack’ the prosurvival PI3K/Akt pathway [23]. Our results show that although ADP lacked most of the apoptosis sequences of apoptin, it retained its ability to interact with AKT. Thus, we hypothesized that ADP inhibits the prosurvival PI3K/Akt pathway and induces apoptosis.

The direct interaction of apoptin with the SH3 domain of the p85 regulatory subunit of PI3K through its proline-rich sequence is necessary for its cytotoxic activity of apoptotic cells [19]. Recent studies by Panigrahi et al. [26] and Jaganmohan et al. [27] suggested that TAT-apoptin not only strongly binds to the SH3 domain of Bcr-Abl but also modifies the phosphorylation state of Bcr-Abl and the activity of its downstream targets. These changes result in the induction of natural apoptosis in myeloid leukemia cells. ADP (via PI3K/Akt signaling) inhibits cell migration and invasion by blocking PI3K activity, reducing p-Akt levels, and downregulating MMP-9 expression in tumor cells (data not shown).

The pathogenesis of GBM is complex, and there are no effective treatments for this disease [28–31]. Aside from surgical excision, temozolomide (TMZ), an orally administered DNA alkylating agent, is the most effective chemotherapy available for GBM patients [2]. However, some patients develop resistance to TMZ, and the overall outcome of GBM patients has not exponentially improved [32]. This is an important finding because GBM therapies fail due to tumor recurrence [33]. Our results show that the anti-cancer activity of the ADP compares favorably with those of temozolomide and rapamycin.

We believe that ADP is the most efficient treatment against GBM tumors because ADP addresses three major challenges in current GBM therapy: (i) Because ADP is smaller than apoptin, it exhibits reduced immunogenicity. *In vivo* and *in vitro* experiments showed that ADP exhibits stronger antitumor activity against GBM. (ii) ADP is based on the reduction of HSP70 in tumor cells targeted with apoptin transformation-derived peptides. This molecule more rapidly reduces HSP70 expression at the mRNA and protein levels, suggesting stronger anti-tumor activity. Thus, this molecule has stronger potential for treating cancer. (iii) Although TMZ greatly improved the clinical treatment of GBM, resistance mechanisms limit its use for long periods of time. The combination of this treatment with ADP might reduce or eliminate drug resistance, thus providing a new method for GBM treatment.

In conclusion, our research explored the apoptin domain region that interacts with the HSE and further showed the anti-cancer effects of the cell-permeable version of ADP in HSP70-expressing human cancer cell lines. Our results provide the first demonstration that the numbers of Ki-67- and GFAP-expressing cells were decreased and increased, respectively, in xenografts upon ADP administration. The treatment also induced cell detachment *in vitro* and increased the sizes of the intercellular spaces *in vivo*. Due to its cytotoxic properties, ADP efficiently reduced disease progression *in vivo*. Indeed, the treatment inhibited tumor xenograft growth and increased the overall health and survival of nude mice implanted with GBM cell lines. These effects were measured in tumors obtained from cell lines and were observed in both intracranial and subcutaneous xenografts.

To conclude, we provide the first demonstration that an apoptin-derived peptide, known as ADP, acts as an effective therapeutic drug in human GBM. This study supports ADP as a potent candidate for drug development and offers the advantages of favorable toxicity and pharmacokinetic profiles. In addition, its use offers time- and cost-saving benefits. Further investigations to determine its underlying mechanisms and its place among the therapeutic strategies for the treatment of GBM are warranted.

## MATERIALS AND METHODS

### Cell lines, patient cohort, polypeptides, and plasmids

The human glioma cell lines U87-MG and U251-MG were obtained from the Department of the Third Affiliated Hospital of Harbin Medical University. Human embryonic kidney (HEK) 293 cells were obtained from the Shanghai Institutes for Biological Sciences Cell Resource Center. Rat glioma cells (C6-RFP) and human normal brain radial glial HA cells were purchased from ATCC (China) and were maintained at 37°C with 5% CO_2_ in complete DMEM (Beijing hiSoft Biological Chemical Co., Ltd., China) supplemented with 15% fetal bovine serum (FBS; Zhejiang Tianhang Biological Technology Co., Ltd., China). pcDNA3.1-SP70,pcDNA3.1plasmids were constructed in our laboratory. The ADP and GST-ADP were synthesized by the Chinese biotechnology company Yaoqiang. Surgical specimens of the primary tumor and the respective in-field tissues were received from the archives of the Department of Pathology at the Daqing Oilfield General Hospital. The use of animals was approved by the Ethic Committees of Harbin Medical University, and all experimental procedures were conducted in accordance with the regulations of the Ethics Committees of Harbin Medical University. Informed consent was obtained from all patients, and the study was approved.

### Immunohistochemistry and HE staining

A morphological analysis of human glioma tissues and BALB/c mouse gliomas through HE staining was performed one week post-injection. All samples were fixed overnight in 4% formaldehyde and embedded into liquid paraffin, and 3-μm sections were then stretched in hot water, mounted onto positively charged slides, and air-dried overnight at 37°C. HE-stained histological sections on the glass slides were used to confirm the presence of tumor tissue. Conventional immunohistochemistry for Ki-67 (ab136912, Abcam, USA), GFAP (ab194326, Abcam, USA), and HSP70 (ab5444, Abcam, USA) was performed. The sections were observed with a light microscope (Olympus, Japan), and statistical analyses were performed.

### Real-time quantitative PCR

The total RNA from U87-MG, U251-MG, and HA cells as well as NBT and GBM tissue was harvested using the TRIzol reagent (Invitrogen, USA) according to the manufacturer's recommended protocol. cDNA synthesis was performed using a High-Capacity cDNA Reverse Transcription Kit (Haigene, China) according to the manufacturer's instructions. The HSP70 mRNA levels were quantified using the Fast SYBR Green Master Mix (Haigene, China) and an ABI 7500 Fast Real-Time PCR System (Applied Biosystems, USA). GAPDH was used as an internal standard for HSP70. PCR was performed using the gene-specific primers NCBI accession number: NM_005345; forward: GCAACGTGCTCATCTTTGA; reverse: TCGCTTGTTCTGGCTGATGT (Jima, China). Each sample was analyzed at least in triplicate. The internal mRNA levels were standardized according to a method described by Applied Biosystems. mRNA expression was represented by the threshold cycle (Ct), and the relative expression level of HSP70 was calculated using the 2^−ΔΔCt^ method.

### Cell viability assay

A 3-(4,5-dimethylthiazol-2-yl)-2,5-diphenyltetrazo-lium bromide (MTT) assay was used to measure glioma cell viability. Briefly, glioma cells (1×10^5^ cells/well) were seeded into a 96-well plate with ADP (China Peptides, China) at final concentrations of 20, 40, 60, 80, and 100 μg/mL or 32 μM RAPA (Tasyl Diyi Pharmaceutical Co., China) for 24 hours. In addition, U87-MG cells were treated with 80 μg/mL ADP, and U251-MG cells were treated with 90 μg/mL ADP, HA (90 μg/mL), or C6 (90 μg/mL) for different amounts of time (12, 24, 36, and 48 hours). Twenty microliters of MTT solution were added to each well, and the cells were incubated for an additional 4 hours. After the medium was discarded, 150 μL of DMSO was added, and the absorbance at 490 nm was recorded.

In addition, apoptosis was detected by flow cytometry using an Annexin V-FITC/propidium iodide (PI) kit (Beyotime, China). Briefly, the cells were pelleted, resuspended in 20 μg/mL Annexin-V-FITC, washed with phosphate-buffered saline (PBS), and stained with 5 μg/mL PI. The samples were subjected to flow cytometry on a BD FACSCanto II (Becton Dickinson), and the data were analyzed using FlowJo software.

### Immunofluorescence and fluorescent imaging

The cells were cultured in DMEM and were treated with ADP (80 μg/mL), apoptin (100 μg/mL), or PBS. After culture for 6, 12, or 24 hours, the cells were fixed in 4% paraformaldehyde in PBS, permeabilized in 0.1% Triton X-100, and stained with either HSP70 (ab5444, Abcam, USA) or TAT antibody (sc-376292, Santa Cruz, USA) and then with their respective secondary antibodies conjugated to Cy3. Twenty milliliters of Hoechst 33342 or DAPI nuclear dye were then added to each well for 20 minutes, and the cells were washed three times with PBS and stained with AO/EB. The fluorescence signal was acquired through multi-laser confocal microscopy.

### GST pull-down assay, protein identification, and immunoprecipitation (IP)

The GST and recombinant GST-ADP proteins were purified using glutathione sepharose beads (Pierce, USA) according to the manufacturer's recommended protocol. A GST pull-down assay was performed to detect the interaction partners of apoptin. Briefly, either purified GST or GST-ADP along with the total U87-MG cell lysate was immobilized on glutathione sepharose beads overnight at 4°C in IP buffer with protease and phosphatase inhibitors. For step-specific actions, refer to a previous study [30].

### Tumor growth experiments

All animal experiments were approved by the Harbin Medical University Subcommittee on Research Animal Care (China) and were performed in accordance with the guidelines and regulations set by the National Institutes of Health. Female 4- to 6-week-old athymic nude mice were anesthetized with a mixture of 100 mg/kg ketamine and 5 mg/kg xylazine in 0.9% sterile saline. U87-MG cells were intracranially injected at a rate of 0.4 μL/minute using a Micro 4 Microsyringe Pump Controller (Hamilton, Switzerland) attached to a syringe with a needle (Gaoge, China) into the mid-left striatum at the following coordinates from the bregma: +0.45 mm anterior-posterior, +2.0 mm mediolateral, and –2.4 mm dorsoventral. Each U87-MG glioma model mouse was injected with approximately 3 × 10^5^ U87-MG cells in 3 μL of DMEM. The animals were examined daily for alertness, motor deficits, and the presence of neurological signs, and their body weights were measured daily. After two weeks, the tumor size was measured by MRI, and the mice were injected with ADP (5 mg/kg/day), PBS alone or TMZ (25 mg/kg/day) via the intraperitoneal route. The mice belonging to the ADP group were injected with 20 μg of bradykinin (BK) in 20 μL of PBS, and 15 minutes later, an injection of 20 mg of ADP in 0.1 mL of PBS was administered. After one to two weeks of continuous ADP administration, the tumor size was measured by MRI. At the end of the experiment, the mice were sacrificed for histopathological analysis. A 3.0-T MRI machine (GE) at Daqing Oilfield General Hospital was used to measure the tumor size through T1 WI, T2 WI, and enhanced T1 WI and T2 WI scans.

For subcutaneous implantation, 5 × 10^5^ cells were subcutaneously injected in the right flanks of the mice. Tumors were apparent one to two weeks after injection. As soon as the subcutaneous tumors reached a volume of 2 to 4 mm^3^, the mice were randomized into two equivalent groups and administered intraperitoneal (i.p.) injections for 15 days of ADP (5 mg/kg) or RAPA (5 mg/kg) as a positive control. The tumor size and general clinical status were recorded every five days and calculated using the following formula: volume (mm^3^) = (length × width^2^)/2. After five weeks of treatment, the mice were euthanized, and the tumors were excised and rinsed in PBS. A 1-mm^3^ portion was fixed in 2.5% glutaraldehyde. The remaining tumor was then fixed in formalin and embedded in paraffin for histological analysis.

### Statistical analysis

Group data are expressed as the mean ± S.E.M. Differences between two groups were assessed using Student's *t*-test. Multiple groups were compared with one-way ANOVA accompanied by Bonferroni's multiple comparison test. *P* < 0.05 indicated statistical significance. Data were analyzed using GraphPad Prism, version 5.0.
